# OTEH-1. Alternative RNA splicing modulates composition of ribosomes and determines spatial phenotype of glioblastoma cells

**DOI:** 10.1093/noajnl/vdab070.040

**Published:** 2021-07-05

**Authors:** Marat Pavlyukov, Tatyana Larionova, Soniya Bastola, Victoria Shender, Ichiro Nakano, Michail Shakhparonov

**Affiliations:** 1 The Institute of Bioorganic Chemistry of Russian Academy of Sciences, Moscow, Russian Federation; 2 University of California, Los Angeles, Los Angeles, CA, USA; 3 Federal Research and Clinical Center of Physical-Chemical Medicine of Federal Medical Biological Agency, Moscow, Russian Federation; 4 Tsukuba University, Tsukuba, Japan

## Abstract

Glioblastoma (GBM) is an extremely heterogeneous tumor and its different regions are populated with phenotypically distinct types of cancer cells. However, it is still unclear how multiple GBM populations arise from the originally homogenous group of tumor precursor cells. Here we showed that GBM cells from the core and edge of the tumor have different composition of ribosomes due to the alternative RNA splicing of multiple ribosomal genes with highest differences observed for RPL22L1. We found that cells at the edge of the tumor express classical isoform of RPL22L1 (RPL22L1a) while core cells have a novel RPL22L1b isoform. RPL22L1b appears due to low pH condition at the core of the tumor. It allows cells to survive during acidosis, promotes more aggressive phenotype in vivo and correlate with worse patient outcome. Mechanistically, RPL22L1b binds to lncRNA MALAT1 in the nucleus and induces its degradation enhancing stemness of GBM cells. On the other hand, RPL22L1a interacts with ribosomes in cytoplasm and upregulates p53 translation favoring less aggressive edge phenotype of GBM. The splicing switch between RPL22L1 isoforms is regulated by SRSF4 proteins. We identified a small molecule compound that inhibits SRSF4 and impairs splicing of RPL22L1, inducing apoptosis of GBM cells and decreasing tumor growth in vivo. Altogether, our data unraveled the mechanism by which less aggressive edge-like GBM cells acquire more malignant core-like phenotype during tumor growth. It may also explain discrepancies between proteome and transcriptome of GBM cell populations. Targeting this pathway may help to decrease tumor heterogeneity and eliminate therapy resistant cells at the tumor core.

Work was supported by the Russian Science Foundation grant 19-44-02027.

